# Stiff-Person Syndrome: Seeing Past Comorbidities to Reach the Correct Diagnosis

**DOI:** 10.1155/2021/6698046

**Published:** 2021-01-31

**Authors:** Jared Hicken, Daniel Ramirez, Mark Rigby, Aram Minasian

**Affiliations:** ^1^Base Medical Student, Ascension Genesys Hospital, Grand Blanc, Michigan, USA; ^2^Department of Internal Medicine, Ascension Genesys Hospital, Grand Blanc, Michigan, USA

## Abstract

Stiff-person syndrome (SPS) is a rare disorder seen in approximately one in one million people. Although it is rare, the symptoms and findings of a typical case should paint a clear clinical picture for those who are familiar with the disease. The primary findings in SPS include progressive axial muscle rigidity as well as muscle spasms. These symptoms most commonly occur in the setting of antibodies against Glutamic Acid Decarboxylase (GAD), the rate-limiting enzyme in the production of Gamma-Aminobutyric Acid (GABA), which is the primary inhibitory enzyme in the central nervous system. Here, we report the case of a 65-year-old African-American female with a past medical history of hypothyroidism, anxiety, and depression with psychotic features who presented with axial muscle rigidity and lactic acidosis. She had been symptomatic for several months and reported extensive workups performed at two previous hospitals without a definitive diagnosis. A complete neurological and musculoskeletal investigation yielded no positive findings except for the presence of GAD antibodies. The patient was treated with diazepam, tizanidine, and Intravenous Immunoglobulin (IVIG) with significant improvement, thus solidifying the diagnosis of SPS, a rare autoimmune and/or paraneoplastic syndrome.

## 1. Introduction

Stiff-person syndrome is a rare autoimmune and/or paraneoplastic syndrome with a prevalence of one in every one million people, although some studies suggest this may be an underestimation [1]. It is characterized by progressive muscle rigidity, most commonly occurring in the axial muscles, although rigidity can also occur in the extremities. In addition to rigidity, the patient will often experience intense, episodic muscle spasms that may be triggered by loud noises, bright lights, or emotional upset [2]. Patients with SPS are most often positive for antibodies against GAD, although more recent studies are finding several other antibodies associated with the disease, including antibodies against GABA type A receptor-associated protein (GABARAP), glycine receptor (GlyR), and glycine transporter 2 (GlyT2) [2–5]. While they are the most commonly seen, antibodies against GAD are not diagnostic of the disease. However, they can, along with the clinical picture, help confirm SPS [6]. Anti-GAD antibodies are occasionally absent, particularly in the case of paraneoplastic SPS, which accounts for approximately ten percent of cases. In paraneoplastic SPS, antibodies against amphiphysin are more common [7–9]. When SPS is seen as a paraneoplastic syndrome, it most commonly occurs in the setting of breast cancer or small cell lung cancer [4].

As GAD is the rate-limiting enzyme in the synthesis of GABA, it is hypothesized that the presence of GAD antibodies may lead to reduced levels of GABA in the CNS. This reduction in GABA, which is the primary inhibitory neurotransmitter in the CNS, would help to explain the common presentation of the disease, decreased nerve inhibition resulting in increased nerve firing, which leads to muscle rigidity and spasms. However, the degree of inhibition is independent of GAD titers, which casts doubt on the causative role of these antibodies in the pathogenesis of the disease [10].

## 2. Case Presentation

The patient was a 65-year-old African-American female with a medical history of recently diagnosed hypothyroidism and chronic anxiety and depression with psychotic features who presented to the emergency department with a chief complaint of “muscle spasms.” She reported these spasms had been occurring on and off for several months and they had been increasing in intensity and frequency. The patient mentioned that the spasms seemed to be triggered by loud noises and bright lights. She reported achy muscle pain all over her body. The patient stated she was recently prescribed levothyroxine. She was otherwise in her usual state of health. She reported recent visits to two nearby hospitals for this same complaint; however, no definitive cause was diagnosed. On physical exam, the patient was diaphoretic and displayed generalized weakness. Her abdominal muscles were hypertonic, but there were no peritoneal signs or masses. There were no other musculoskeletal abnormalities. Labs were drawn, and the patient was given IV lorazepam with improvement. Initial labs were significant for creatine kinase 372 IU/L (38–234 IU/L), lactate dehydrogenase 363 IU/L (98–192 IU/L), lactic acid 4.50 mmol/L (0.50–2.20 mmol/L), thyroid-stimulating hormone 12.820 ulU/ml (0.270–4.20 ulU/ml), and leukocytosis at 13.1 K/cmm (4.5–11.0 K/cmm). These lab findings prompted admission to the hospital for further workup.

Chest X-ray performed upon admission did not show evidence of any acute cardiopulmonary processes. However, she was positive for “left lower lobe subsegmental atelectasis and/or scarring.” This was considered an incidental finding. An X-ray of the abdomen showed nonobstructive bowel gas patterns. Lactic acid normalized on hospital day two, dropping to 1.83 mmol/L; however, there was no other improvement in lab values and the patient had not made significant clinical improvements. Other findings during her hospitalization included dysphagia and regurgitation when eating solids or liquids. A formal speech evaluation was performed which the patient immediately failed, prompting a gastroenterology consult for esophagogastroduodenoscopy. This evaluation revealed sluggish to absent motility of the esophagus.

On hospital day 3, GAD antibody was tested and came back at >250 IU/mL (0.0–5.0 IU/mL). This finding, along with the clinical presentation of the patient, led to a diagnosis of SPS. The patient was then started on diazepam and tizanidine. Upon making the diagnosis, our immediate concern was to rule out malignancy. Although paraneoplastic SPS is generally negative for GAD antibodies, this is not always the case [11]. CT neck/chest/abdomen/pelvis was ordered, which showed no signs of malignancy. The patient subsequently received a course of IVIG for 5 days and reported significant improvement with this treatment. The patient was admitted to inpatient rehab where she continued physical and occupational therapy. After 10 days of therapy, the patient was discharged home in stable condition.

## 3. Discussion

Although the patient presented with very typical symptoms for SPS, she also had a number of comorbid conditions that likely played a role in delaying diagnosis. For one, she had a significant history of anxiety and depression with psychotic features. It does not seem unreasonable to believe that her psychiatric history could have played a role in her presentation. Additionally, the patient had recently been diagnosed with hypothyroidism and had an elevated TSH level upon presentation to the emergency department. While her symptoms would seem extreme for hypothyroidism, low thyroid hormone levels can lead to symptoms such as fatigue, arthralgia, and myalgia. However, it is important to note that anxiety is a common result of SPS and comorbid autoimmune conditions, such as hypothyroidism, are also common [1, 2].

The differential diagnosis for this patient was quite broad. Among the other diagnoses considered were ankylosing spondylitis (AS), tetanus, and Parkinson's disease. AS can also present with truncal stiffness, but there were no signs on imaging of sacroiliitis or spondylitis. Tetanus can present with trunk and limb spasms similar to those seen in SPS, but commonly has trismus and facial spasms, which were not seen in the patient. Parkinson's disease was ruled out based on physical exam, where cogwheel rigidity and resting tremor were absent. Overall, the patient's symptoms lined up quite well with the diagnostic criteria for stiff-person syndrome, as outlined by Baizabal-Carvallo et al., shown in Table 1 [7].

Although the finding of esophageal dysmotility initially seemed unrelated to the patient's diagnosis, further consideration of the hypothesized physiology of SPS made it reasonable to assume this finding was actually a result of the disease. Per review by Witte et al., in 150 cases of SPS that had been reported at the time of their publication, 6 had reported pharyngeal or esophageal dysphagia. Follow-up esophageal manometry revealed the first case of esophageal dysmotility that was objectively confirmed in an SPS patient [12]. This finding suggested that SPS can affect both striated and smooth muscle.

Upon diagnosis of our patient, various treatment methods were used to improve the patient's condition. The patient was initially started on diazepam, a GABA-enhancing drug, and tizanidine, an antispastic agent. Both of these are frequently recommended as options for initial treatment, as outlined by Dalakas et al., shown in Table 2 [13]. Due to the severity of the patient's symptoms, the decision was made to additionally initiate treatment with intravenous immunoglobulin. The patient experienced significant improvement with these therapies, though she was not completely asymptomatic afterwards.

## 4. Conclusions

This patient presented with symptoms that were very typical for SPS; however, her comorbid conditions seemed to have previously explained some of her symptoms. This diagnosis has been historically difficult to establish and is often misdiagnosed, as stated in previous reviews [1, 2]. A high index of suspicion should be held in patients with recurrent episodes of axial rigidity and spasms, and providing awareness of this uncommon pathology may help eliminate delay of diagnosis and the waste of medical resources and provide rapid quality care to suffering patients with SPS.

## Figures and Tables

**Table 1:** Diagnostic criteria of stiff-person syndrome [7].

Diagnostic criteria of stiff-person syndrome
(i) Stiffness in the axial and limb muscles resulting in impairment of ambulation
(ii) Presence of superimposed episodic spasms that are precipitated by sudden movement, noise, or emotional upset
(iii) A positive therapeutic response to oral diazepam or findings of continuous motor-unit activity on electromyography (EMG) that are abolished by intravenous diazepam
(iv) Absence of other neurologic disorders that may explain the clinical features

**Table 2:** Most commonly used therapies for patients with stiff-person syndrome [13].

Most commonly used therapies for patients with stiff-person syndrome		
Therapy	Dose per day	Presumed mechanism of action
*GABA-enhancing drugs*		
Sedative anxiolytics		
Diazepam	5–100 mg	Central GABA_A_ agonist
Clonazepam	2.5–6 mg	Central GABA_A_ agonist
Alprazolam	2–4 mg	Central GABA_A_ agonist
Lorazepam	6 mg	Central GABA_A_ agonist
Antiepileptic drugs		
Vigabatrin	2–3 g	Irreversible inhibition of GABA-transaminase
Valproate	0.6–2 g	Augments GABA transmission
Gabapentin	3600 mg	Structurally related to GABA, but the mechanism of action is unknown
Levetiracetam	2000 mg	Facilitates inhibition of GABAergic transmission
Tiagabine	6 mg	Blocks GABA reuptake

*Antispasticity agents*		
Baclofen	10–60 mg	GABA_B_ agonist
Tizanidine	6 mg	Central *α*2-adrenergic action; inhibits norepinephrine release
Dantrolene	200–400 mg	Dissociates excitation-contraction coupling and blocks release of Ca^++^ from the sarcoplasmic reticulum
Botulinum toxin A	—	NMJ blocking; prevents acetylcholine exocytosis

*Immunotherapies*		
IV immunoglobulin	2 g/kg	Immunosuppression/modulation
Rituximab	2 g (in two divided doses)	B-cell depletion
Plasmapheresis	5–6 passes	Immunosuppression/modulation
Corticosteroids	Up to 60 mg	Immunosuppression/modulation
Immunosuppressive agents		
Azathioprine	2.5–3 mg/kg	Immunosuppression/modulation
Methotrexate	15–20 mg	Immunosuppression/modulation
Mycophenolate	2–3 g	Immunosuppression/modulation

^*∗∗*^GABA = *γ*-aminobutyric acid, IV = intravenous, NMJ = neuromuscular junction		

## Data Availability

No datasets were generated or analyzed during the current study.

## References

[1] Galli J. R., Austin S. D., Greenlee J. E., Clardy S. L. (2018). Stiff person syndrome with anti-GAD65 antibodies within the national veterans affairs health administration. *Muscle & Nerve*.

[2] Alexopoulos H., Dalakas M. C. (2010). A critical update on the immunopathogenesis of stiff person syndrome. *European Journal of Clinical Investigation*.

[3] Sarva H., Deik A., Ullah A., Severt W. L. (2016). Clinical spectrum of Stiff person syndrome: a review of recent reports. *Tremor and Other Hyperkinetic Movements*.

[4] Balint B., Bhatia K. P. (2016). Stiff person syndrome and other immune-mediated movement disorders-new insights. *Current Opinion in Neurology*.

[5] Pittock S. J., Lucchinetti C. F., Parisi J. E. (2005). Amphiphysin autoimmunity: paraneoplastic accompaniments. *Annals of Neurology*.

[6] Meinck H.-M., Thompson P. D. (2002). Stiff man syndrome and related conditions. *Movement Disorders*.

[7] Baizabal-Carvallo J. F., Jankovic J., Joseph J. (2014). Stiff-person syndrome: insights into a complex autoimmune disorder. *Journal of Neurology, Neurosurgery & Psychiatry*.

[8] Mckeon A. (2012). Stiff-man syndrome and variants. *Archives of Neurology*.

[9] Ariño H., Höftberger R., Gresa-Arribas N. (2015). Paraneoplastic neurological syndromes and glutamic acid Decarboxylase antibodies. *JAMA Neurology*.

[10] Raju R., Foote J., Banga J. P. (2005). Analysis of GAD65 autoantibodies in stiff-person syndrome patients. *The Journal of Immunology*.

[11] Sarwari N., Galili Y., Perez A., Avgeropoulos N., Tseng J. (2019). Unusual presentation of lung adenocarcinoma with paraneoplastic Stiff person syndrome: role of EGFR tyrosine kinase inhibitors. *Journal of Thoracic Oncology*.

[12] Witte T. N., Borum M. L., Ali M. A. (2006). Esophageal dysmotility confirmed by esophageal manometry in a patient with stiff-man syndrome and dysphagia. *American Journal of Gastroenterology*.

[13] Dalakas M. C. (2009). Stiff person syndrome: advances in pathogenesis and therapeutic interventions. *Current Treatment Options in Neurology*.

